# A Centralized AI Lakehouse Framework for Brain Tumor MRI Classification and Segmentation, University KPI Forecasting, and Water Potability Prediction

**DOI:** 10.3390/s26123804

**Published:** 2026-06-15

**Authors:** Ronish Shrestha, Md Masud Rana, Bo Sun, Frank Sun, Helen Lou, Alek Hutson

**Affiliations:** 1 Department of Computer Science, Lamar University, Beaumont, TX 77705, USA; rshrestha14@lamar.edu (R.S.); bsun@lamar.edu (B.S.); sunqx@lamar.edu (F.S.); 2 Center for Data, AI and Cybersecurity (CDAC), Lamar University, Beaumont, TX 77705, USA; hhlou@lamar.edu; 3 Center for Midstream Management and Science (CMMS), Lamar University, Beaumont, TX 77705, USA; ahutson@lamar.edu

**Keywords:** AI lakehouse, water potability, random forest, KPI forecasting, ARIMA, brain tumor, MRI segmentation, DeepLabV3, FastAPI, Docker

## Abstract

In many university and healthcare projects, models are built for very different data types such as tables, institutional time series, and medical images, but they are deployed as separate applications. In this work, that separation made testing and maintenance difficult because each module had its own pipeline and runtime requirements. This paper presents an integrated AI lakehouse-style implementation that runs three model pipelines inside one containerized backend. For medical imaging, we used MRI datasets from IEEE DataPort: a four-class classification set with 7012 images (5708 train/1304 test) and a segmentation set with 3063 image–mask pairs. The classification model (ResNet50 transfer learning) is evaluated using a proper train–validation–test protocol across multiple splits (80/10/10, 70/10/20, 60/10/30, and 10/30/60), achieving a test accuracy of 99.00% under the standard 80/10/10 split. Additionally, a patient-level evaluation is conducted using an external glioma dataset to provide a more realistic assessment without data leakage. The segmentation model (DeepLabV3-ResNet50) achieved 83.09% validation mIoU and 88.79% Dice score. For university KPI forecasting, we used annual IPEDS and NSF HERD data from 2010 to 2023 for three universities (BSU, EOU, and UAB). To examine the effect of preprocessing on forecasting performance, two case studies are conducted. In the first case, linear interpolation is applied to generate semester-level data. In the second case, the original annual data is used directly without interpolation. Random Forest regression and ARIMA models are evaluated using MAE, RMSE, MAPE, and $R^{2}$. The results showed that interpolation improved apparent forecasting performance due to smoothing, while evaluation on the original annual data provided a more realistic assessment of model behavior. To further validate the framework on a larger dataset, an additional case study is conducted using a student dropout dataset. For water potability, we trained and compared multiple tabular classifiers on a large dataset (1,048,575 samples). A Random Forest model (100 trees, max depth 10) achieved 85.86% test accuracy and high recall for unsafe samples (0.8447). All modules are served via FastAPI and deployed together using Docker, with workflow automation routing requests to the correct endpoint. System-level benchmarking indicates that the backend maintains stable throughput and latency under concurrent requests.

## 1. Introduction

Modern applications generate large amounts of data from different sources, including structured databases, time-series records, and image-based systems. Machine learning and deep learning models have shown strong performance in analyzing these data types. However, in practice, these models are often developed and deployed independently for each domain. This leads to separate pipelines, different runtime environments, and increased system complexity.

In real-world settings, this fragmented approach makes it difficult to manage and scale AI systems. Each model requires its own preprocessing steps, dependencies, and deployment configuration. As a result, maintaining multiple AI systems becomes challenging, and reproducibility across environments is reduced. This creates a need for a single system that can manage different types of AI workloads in a consistent and efficient way [1].

To address this issue, this study proposes an operational AI Lakehouse framework for integrated deployment and orchestration of heterogeneous AI workloads. The framework focuses on system-level integration, where multiple AI models are deployed, managed, and executed within a single backend environment. The goal is not to combine these models into a single predictive task, but to provide a consistent and manageable system for handling different types of AI workloads.

**Figure 1:**
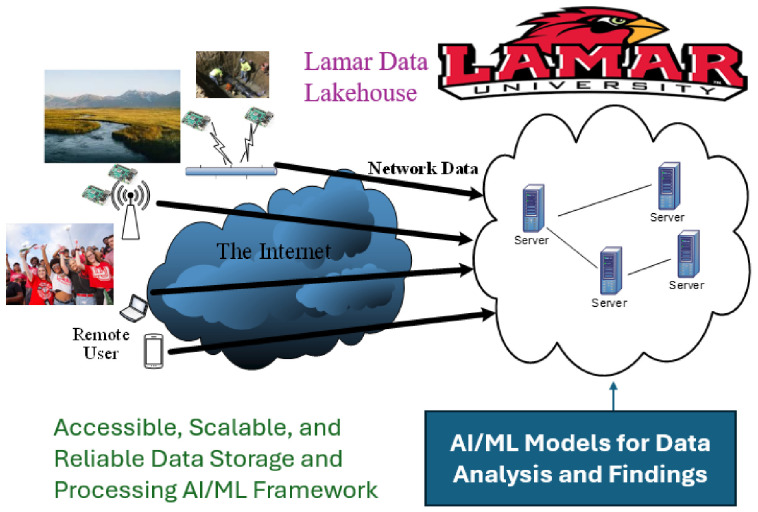
Conceptual overview of the Lamar Data Lakehouse framework illustrating data acquisition from heterogeneous sources, network-based transmission, centralized storage infrastructure, and integrated AI/ML-driven analysis.

Figure 1 shows the conceptual structure of the Lamar Data Lakehouse framework used in this study. In modern environments, data is collected from multiple sources such as institutional databases, sensors, user interactions, and remote systems. These data streams are transmitted through network infrastructure and stored in centralized servers.

Traditional data pipelines mainly focus on storage and analytics. However, integrating AI models into these pipelines is still difficult due to differences in data formats, model requirements, and deployment setups. As a result, AI systems are often built as separate components, which increases system complexity and reduces maintainability.

The proposed framework extends the traditional data pipeline by incorporating AI model execution within the same system. In this architecture, data ingestion, storage, processing, and model execution are handled within a single backend environment. Incoming data is routed to appropriate AI modules based on its type, including image-based models, time-series forecasting models, and tabular machine learning models.

A key component of the framework is the use of n8n for workflow orchestration. It provides a centralized routing layer that directs requests to different AI modules through a single entry point. This simplifies system interaction and reduces deployment complexity. The contribution of this work is therefore system-oriented, focusing on deployment and orchestration rather than combining different domains into a single model.

Different machine learning models are suited for different types of data. For structured tabular data, Random Forest is widely used due to its ability to model complex relationships [2]. For time-series forecasting, models such as ARIMA are effective for short sequences as they capture temporal trends with fewer data points [3]. In medical imaging, deep learning models such as ResNet50 and DeepLabV3 are commonly used for classification and segmentation tasks [4,5].

Although these models perform well in their respective domains, integrating them into a single system remains a challenge. Each model typically requires different preprocessing pipelines and dependencies, which increases operational overhead [1]. Recent work in data lakehouse architectures has focused on unifying data storage and analytics [6]. Building on this idea, this study extends the concept to support integrated AI model deployment and execution.

**Figure 2:**
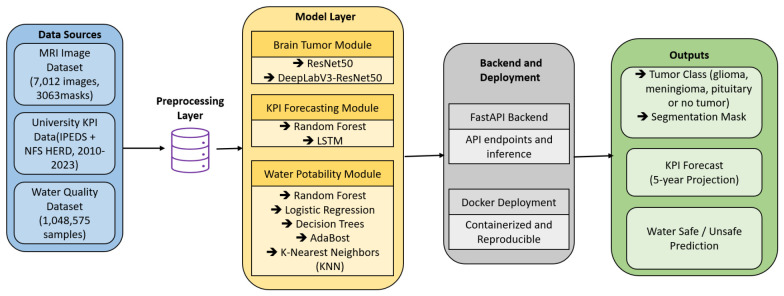
Proposed lakehouse data pipeline architecture, including data extraction, transformation, storage, and integrated AI/ML processing.

Figure 2 illustrates a general data pipeline where heterogeneous data is extracted, transformed, and stored before being used for analytics and AI tasks. In conventional systems, AI components are often deployed separately. In contrast, this work integrates multiple AI modules within a single backend framework to represent heterogeneous workloads.

As a proof of concept, three representative modules are implemented. In addition, an extra case study is included to further evaluate the framework on a larger dataset:

1. Brain Tumor MRI Analysis: A medical imaging module that performs tumor classification using ResNet50 and segmentation using DeepLabV3–ResNet50. To improve evaluation reliability, both image-level and patient-level validation strategies are considered.
2. University KPI Forecasting: A time-series forecasting module using Random Forest regression and ARIMA models to analyze institutional trends.
3. Water Potability Prediction: A tabular classification module using multiple machine learning models, with Random Forest selected as the final deployed model.

An additional student dropout case study is also included to evaluate the framework on a larger dataset and in a different problem setting. These modules are integrated within a single FastAPI backend and deployed using Docker. This ensures consistent execution, simplified management, and improved reproducibility across different environments [1]. The main contribution of this work lies in demonstrating how heterogeneous AI workloads can be integrated and deployed within a single operational system.

## 2. Related Work

Many studies have applied machine learning and deep learning to solve problems in medical imaging, time-series forecasting, and structured data analysis (Table 1). However, most of these works focus on improving the model performance in a single domain. Fewer studies explore how different types of AI models can be deployed together within a single integrated system. Additionally, integrated systems that support deployment and execution of multiple AI models for both structured data and image analysis within a single backend are not commonly available.

### 2.1. Lakehouse and Integrated Data Architectures

Modern data platforms aim to integrate the flexibility of data lakes with the organization of data warehouses. Armbrust et al. [6] introduced the Lakehouse architecture to support analytics and machine learning on a single platform. Stonebraker et al. [11] also talked about the limitation of traditional database systems and emphasized the importance of modern architectures. However, these studies do not show how different AI models can be deployed on a single backend system.

### 2.2. Deep Learning for Medical Image Analysis

Deep learning has significantly improved medical image classification and segmentation. U-Net [12] and DeepLabV3 [5] are widely used for segmentation tasks. ResNet [4] is commonly used for image classification through transfer learning. The BraTS dataset [10] provides a benchmark for brain tumor segmentation. These studies focus on model performance but do not show how imaging models can be deployed together with tabular and forecasting systems in one framework.

### 2.3. Time-Series Forecasting with LSTM

Research on time series forecasting has been conducted for a long time [13]. LSTM networks were introduced to deal with the problem of long-term dependencies in sequential data [9], and subsequent improvements improved their learning ability [14]. Although ARIMA models are effective for forecasting, most studies focus on improving prediction accuracy rather than system-level integration.

### 2.4. Water Quality Prediction Using Machine Learning

Machine learning has been widely used for water quality prediction. Palani et al. [7] applied neural networks for environmental forecasting. Singh et al. [8] compared various machine learning algorithms for water assessment and concluded that ensemble methods perform well. Random Forest [2] is commonly used for tabular data as it addresses nonlinear relationships and prevents overfitting. However, these studies focus only on water prediction and do not address integration with other AI tasks.

Overall, existing research primarily focuses on improving individual algorithms within specific domains, while comparatively less attention has been given to system-level approaches that integrate and manage heterogeneous AI workloads—such as medical imaging, forecasting, and tabular prediction—within a single backend environment.

### 2.5. Research Gap

Most previous studies either focus on data platform design without implementing complete AI pipelines, or focus on a single task such as water quality prediction, time-series forecasting, or medical image segmentation. Limited work has been done to demonstrate how these different AI tasks can be deployed together within one containerized backend system.

In this study, we address this gap by integrating deep learning based on MRI, KPI forecasting, and tabular prediction into a single FastAPI application that is deployed using Docker. This approach demonstrates how multiple AI workloads can operate together under one consistent and reproducible framework.

## 3. Methods: AI Lakehouse Framework for Brain Tumor MRI Classification and Segmentation, University KPI Forecasting and Water Potability Prediction

### 3.1. Brain Tumor MRI Analysis Module

The brain tumor module was developed using MRI datasets available from IEEE DataPort. The dataset is used in two different forms, one for classification and the other for segmentation.

The brain tumor classification dataset is structured in a folder-based structure, where the dataset is divided into two folders, one for the Training set and the other for the Testing set. Inside the folders, there are four subfolders, each corresponding to the types of tumors, glioma, meningioma, pituitary, and notumor.

In total, the dataset includes 7012 images, where the training set includes 5708 images, out of which glioma includes 1320, meningioma includes 1338, pituitary includes 1456, and notumor includes 1594 images. On the other hand, the testing set includes 1304 images, out of which glioma includes 300, meningioma includes 300, pituitary includes 300, and notumor includes 404 images.

**Figure 3:**
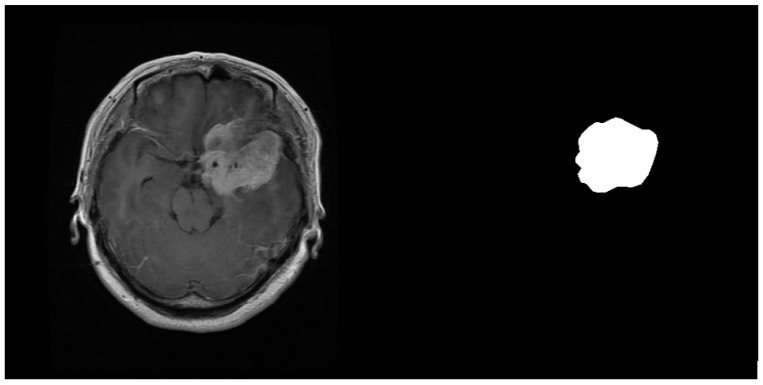
Example MRI image and corresponding tumor mask from the dataset.

For segmentation, the dataset is composed of 3063 paired images of MRI and tumor masks. The tumor masks highlight the tumor areas in the image, represented as the background, making it a binary segmentation problem, as shown in Figure 3.

#### 3.1.1. Patient-Level MRI Evaluation

To address the limitation of image-level splitting, an additional patient-level evaluation was conducted using a glioma MRI dataset from the TCIA UTSW glioma collection [15]. In this dataset, each patient contains multiple MRI slices along with corresponding tumor segmentation masks.

Unlike the classification dataset, where images are treated independently, this experiment ensures that all slices belonging to a single patient are kept within the same data split. This prevents overlap between training and testing samples and avoids data leakage.

Each 3D MRI volume was processed into 2D slices, and slices were labeled as tumor or non-tumor based on the corresponding segmentation mask. Only valid image-mask pairs with matching dimensions were used. Patients with inconsistent image and mask shapes were excluded during preprocessing.

To maintain consistency with the classification experiments, multiple patient-level train–validation–test splits were evaluated, including 80/10/10, 70/10/20, 60/10/30, and 10/30/60. In all cases, splitting was performed at the patient level.

A ResNet50-based classifier with transfer learning was used for this experiment, following the same training setup as the image-level model.

This evaluation provides a more realistic assessment of model performance, as it reflects practical scenarios where predictions are made on entirely unseen patients.

#### 3.1.2. Preprocessing and Data Augmentation

All images were resized before training. For the classification task, images were scaled to 224×224 pixels to fit the input size expected by ResNet50 [4]. The images were resized to 512×512 pixels for the segmentation task to retain more spatial information for precise tumor boundary detection.

To match the distribution of the pretrained ImageNet weights, pixel intensities were normalized. During training, basic data enhancement methods such as random horizontal flipping and small rotations were applied to improve the robustness of the model and reduce overfitting [16].

#### 3.1.3. Brain Tumor Classification Model

For the tumor classification task, we used a ResNet50 model with transfer learning [4]. The network was initialized with pretrained ImageNet weights, and the final fully connected layer was modified to produce four output classes.

The dataset was divided into training, validation, and testing subsets to ensure proper evaluation. The validation set was used during training for model selection, while the test set was kept completely unseen and used only for final performance evaluation.

To further assess robustness, additional experiments were conducted using different train–validation–test splits, including 80/10/10, 70/10/20, 60/10/30, 10/30/60, and an extreme 1/1/98 configuration.

Training was carried out on the Lamar University server using the AdamW optimizer with a learning rate of $5\times 10^{-5}$. Early stopping was applied to reduce the risk of overfitting, and the model was trained for 20 epochs.

The detailed performance metrics for training, validation, and testing are presented in the Results section. The model demonstrates strong performance under standard splits and shows expected degradation when the training data is significantly reduced. Slightly lower performance for the meningioma class may be attributed to visual similarities between tumor types in MRI images.

#### 3.1.4. Brain Tumor Segmentation Model

For tumor segmentation, we used a DeepLabV3-ResNet50 architecture [5]. The task was formulated as a binary pixel-level classification problem, distinguishing tumor regions from background.

All segmentation images were resized to 512×512 before training. Since most MRI slices contain a large number of background pixels compared to tumor pixels, a class-weighted loss function was applied to reduce the effect of class imbalance.

Model performance was evaluated using pixel accuracy, mean Intersection over Union (mIoU), and Dice score. Although pixel accuracy can be high due to the dominance of background pixels, mIoU and Dice score provide a more reliable measure of how well the predicted tumor regions match the ground-truth masks [10,12].

The final validation results are shown below:

- Validation Loss: 0.0779
- Pixel Accuracy: 99.30%
- Validation mIoU: 83.09%
- Dice Score: 88.79%

The mIoU and Dice score results clearly show a high degree of agreement between the predicted segmentation masks and actual tumor areas.

(1) $$Dice=\frac{2|A\cap B|}{\left|A|+|B\right|}$$

where *A* and *B* represent the predicted and ground-truth segmentation masks, respectively [17].

#### 3.1.5. Model Deployment

Finally, the classification model and segmentation model were saved as PyTorch 2.10.0 files and integrated into the FastAPI backend for real-time inference within the proposed framework.

### 3.2. University KPI Forecasting Model

The KPI forecasting module builds upon our earlier work [18], where multivariate forecasting models such as Random Forest and ARIMA were applied to institutional time-series data. In that work, the focus was primarily on forecasting performance within a single application setting.

In this study, the role of the KPI module is different. Instead of functioning as a standalone forecasting tool, it is incorporated into the proposed AI Lakehouse framework. The goal here is to demonstrate how time-series models can be deployed and managed alongside other AI components within a single system.

Within the proposed architecture, the KPI module represents the time-series component and supports institutional trend analysis and forecasting. It operates together with the medical imaging and tabular prediction modules, with all components coordinated through n8n-based workflow orchestration.

The KPI dataset originally consisted of annual observations from 2010 to 2023, which provided only 14 data points for each institution. Such short time series limit the effectiveness of complex sequence models. To improve temporal granularity, linear interpolation was applied to generate semester-level observations (Spring, Summer, and Fall) between consecutive annual records. This expanded each institutional series while preserving the overall yearly trend. Based on the experiments, ARIMA provided more stable forecasting performance than LSTM for this dataset, so ARIMA was adopted in the revised KPI analysis.

To address the concern that interpolation may artificially smooth institutional time series, the KPI analysis was extended into two separate case studies. The first case uses interpolated semester-level data to increase temporal resolution, while the second case uses the original annual data without interpolation. This allows direct comparison between smoothed and real-world time series behavior.

The KPI module in this study is used as a case-study component within the proposed framework rather than as a standalone contribution focused on forecasting performance. The dataset consists of annual observations from 2010 to 2023, resulting in only 14 data points per university. This limited size makes it difficult for models, especially sequence-based approaches, to learn stable temporal patterns.

As a result, the KPI forecasting results should be interpreted in the context of demonstrating how different model types can be integrated and executed within a single backend system. The primary contribution of this work lies in the system-level orchestration and deployment of heterogeneous AI workloads, rather than in achieving state-of-the-art forecasting accuracy.

The results show that models, particularly ARIMA, perform more consistently on interpolated data due to reduced noise and smoother temporal patterns. However, performance on the original annual dataset is lower, reflecting the challenges of forecasting with limited observations. This comparison highlights that improvements observed with interpolation are largely due to data smoothing rather than true predictive capability. Therefore, results from the original dataset provide a more realistic evaluation of model behavior.

#### 3.2.1. Datasets and KPI Targets

Annual institutional data was collected from publicly available and standardized sources to ensure transparency and reproducibility:

(i)  Integrated Postsecondary Education Data System (IPEDS) [19]: enrollment, employee counts, and graduates
(ii) National Science Foundation Higher Education Research and Development (NSF HERD) survey [20]: research and development (R&D) expenditure

Four key performance indicators (KPIs) were considered:

(i)    Total enrollment
(ii)   Total employees
(iii)  Total graduates
(iv)  R&D expenditure

The range of the dataset is the years from 2010 to 2023. With the limited amount of data in the dataset, the forecasts should be interpreted in an exploratory manner rather than as conclusive results. This is particularly true with the ARIMA model, which requires more data in time series.

In determining the applicability of the system in different institutions with different sizes, three universities were chosen: Boise State University (BSU), Eastern Oregon University (EOU), and the University of Alabama at Birmingham (UAB).

All experiments were conducted using historical data from 2010 to 2023. No future observed values were used in model training or evaluation. Any values beyond 2023 correspond only to model predictions.

#### 3.2.2. Data Preprocessing and Feature Construction

The raw IPEDS and NSF HERD data were first organized into annual institutional KPI tables covering the years 2010 to 2023. Since each university had only 14 annual observations, two preprocessing settings were considered.

Case Study 1: Interpolated Semester-Level Data. In the first case, linear interpolation was applied to generate semester-level values (Spring, Summer, Fall) between consecutive annual records. For the first year, values were repeated across semesters. For subsequent years, intermediate values were generated using one-third and two-thirds interpolation. This increased the number of observations from 14 annual points to 42 semester-level points per university (see Supplementary Table S1).

Case Study 2: Original Annual Data. In the second case, the original annual dataset was used directly without any interpolation. This preserves real year-to-year variation and provides a more realistic representation of institutional trends.

For Random Forest, lag-based features were constructed. For ARIMA, the series was modeled directly as a univariate time series. Both approaches were evaluated under identical conditions in each case study.

#### 3.2.3. Forecasting Models

Two forecasting approaches were applied and compared in this study:

1. Random Forest Regression [2]: A tree-based ensemble regression method that uses lagged observations and related KPI variables to learn nonlinear patterns. It is suitable for structured institutional data and provides stable performance without requiring long sequential histories.
2. ARIMA [3]: A classical autoregressive integrated moving average model designed for time-series forecasting. ARIMA is particularly suitable for short and trend-driven institutional series because it models temporal dependence directly and does not require large training sequences.

Each model was trained independently for every KPI and evaluated across the three selected universities.

#### 3.2.4. Evaluation Strategy

To maintain the time order of the data, an 80/20 chronological split was applied. The earlier years were used for training, while the most recent years were kept for validation. This approach reflects real forecasting practice and helps prevent data leakage.

The performance of the model is evaluated on the basis of standard regression metrics for time series data [3]:

- Mean Absolute Error (MAE)
- Root Mean Squared Error (RMSE)
- Mean Absolute Percentage Error (MAPE)
- Coefficient of Determination ($R^{2}$)

After model evaluation, multi-step forecasts were generated for future institutional planning periods. These forecasted values were used only for exploratory analysis and do not represent observed data. These forecasts were examined to compare stability, prediction error, and trend behavior across universities of different sizes. The evaluation was conducted separately for both case studies. This enables direct comparison of model performance under interpolated (smoothed) and original (real) data conditions.

#### 3.2.5. Additional Case Study: Student Dropout Prediction

To further evaluate the applicability of the proposed framework on a larger dataset, an additional case study was conducted using a publicly available student dropout dataset from Kaggle [21].

The dataset contains 10,000 student records with features including demographic information, academic performance, attendance, and behavioral factors. The target variable represents whether a student has dropped out or not, making this a binary classification problem.

Missing numerical values were handled using median imputation, while categorical variables were filled using the most frequent value and encoded into numerical form. A Random Forest classifier was used, consistent with the approach used in the water potability module.

To address class imbalance, class weighting was applied during training. The dataset was split into training and testing sets using an 80/20 ratio. The results of this case study are presented in the Results section.

### 3.3. Water Potability Prediction Module

The water potability module is built on structured data from a publicly available water quality dataset [22], where the dataset is represented by different chemical and physical properties of the water sample, as well as a binary label, where 0 represents safe water, while 1 represents unsafe water. The dataset is made of 1,048,575 total samples.

The water potability model is represented by the following features: pH, iron concentration, nitrate level, chloride content, turbidity, sulfate, and total dissolved solids.

To evaluate the generalization of the model, the dataset is split into the training set (80%) and the test set (20%) using stratified sampling, where the class distribution is preserved in the two sets.

#### 3.3.1. Feature Selection and Data Preprocessing

A selected set of relevant water quality features was used for model training to keep the input structure consistent during both training and deployment.

Since environmental datasets often contain missing values due to sensor errors or incomplete reporting, median imputation was used to fill in missing entries. The median was chosen because it is less affected by extreme values compared to the mean [23].

After handling missing values, the features were standardized to support stable model training [24]. All preprocessing steps and model training were organized within a scikit-learn Pipeline to reduce the risk of data leakage and ensure reproducibility.

#### 3.3.2. Model Training and Comparative Evaluation

Several supervised classification algorithms were evaluated:

(i)    Random Forest [2]
(ii)   CatBoost [25]
(iii)  XGBoost [26]
(iv)  Decision Tree [27]
(v)   Logistic Regression [28]
(vi)  AdaBoost [29]

All the models were trained using the same preprocessing techniques, and the test set was used for evaluation. Although several performance metrics were explored, the accuracy and F1-score were used in the final evaluation of the class imbalance situation. The best model in terms of overall performance was the Random Forest model, which was chosen for deployment.

The final model is the Random Forest model, where the number of trees was set at 100, the maximum depth of the tree was set at 10, the training was parallelized over the available CPU cores (n_jobs = −1), and the random state was set at 42.

#### 3.3.3. Final Model Performance

The selected Random Forest classifier achieved the following results on the test dataset:

- Accuracy: 85.86%
- Precision: 0.6472
- Recall: 0.8447
- F1-score: 0.7329

The high value of recall is especially relevant in this problem, as failing to identify unsafe water samples may have public health consequences. Upon training the model, the model was saved and deployed using the FastAPI backend.

## 4. System Architecture and Deployment Framework

### 4.1. AI Lakehouse Design

The primary contribution of this work lies in the system-level integration and orchestration of heterogeneous AI workloads within a single backend environment. Instead of deploying separate pipelines for each task, the proposed framework enables multiple AI models—including medical imaging, time-series forecasting, and tabular prediction—to be executed through a single operational system.

A key feature of the framework is the use of a centralized API layer combined with workflow orchestration, which allows all modules to be accessed through a single entry point. This design simplifies deployment, reduces system complexity, and ensures consistent execution across different data modalities.

In addition, the framework is evaluated from an operational perspective through system-level benchmarking. The results demonstrate that the backend maintains stable throughput and bounded latency under concurrent requests, indicating that the system is not only functionally integrated but also operationally viable.

### 4.2. Overall Architecture

The system is organized into four functional layers:

1. Data Layer: It includes structured data in a tabular form, such as water and KPI, as well as unstructured data for MRI image inputs.
2. Model Layer: It includes trained machine learning, statistical forecasting, and deep learning models. The models are trained for water prediction, KPI forecasting, and brain tumor classification and segmentation, where the models are a Random Forest classifier for water quality, Random Forest regression and ARIMA for KPI forecasting, and ResNet50 and DeepLabV3-ResNet50 for MRI classification and segmentation, respectively.
3. API Layer: It includes FastAPI endpoints for user inputs and model predictions.
4. Deployment Layer: It uses Docker for portability, reproducibility, and dependency consistency.

**Figure 4:**
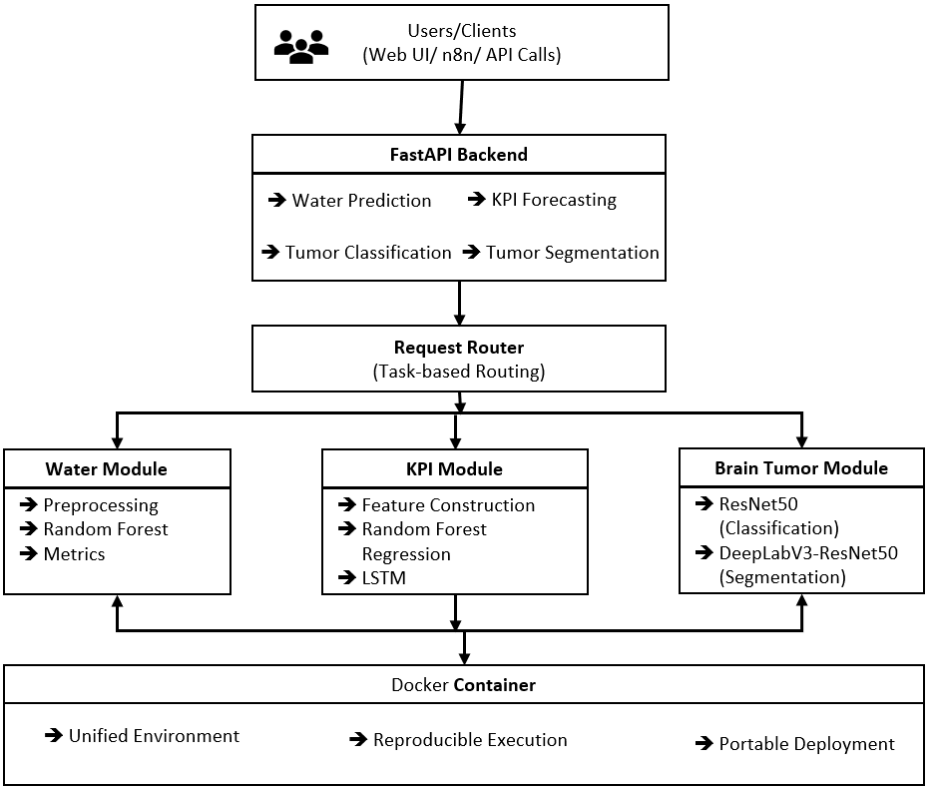
AI system architecture integrating brain tumor analysis, KPI forecasting, and water potability prediction under a centralized FastAPI service and Docker-based deployment.

The overall system architecture is illustrated in Figure 4.

### 4.3. System-Level Benchmarking Setup

To evaluate the runtime performance of the proposed AI Lakehouse framework, a system-level benchmark was conducted on the deployed FastAPI backend. The benchmark was executed for 60 s using 4 concurrent clients and 1 MiB object size under a round-robin configuration.

Separate tests were performed for GET, PUT, and mixed workloads to measure system throughput (objects per second) and request latency (milliseconds). This evaluation focuses on quantifying the operational behavior of the backend under concurrent access.

In addition to throughput and latency measurements, system-level monitoring metrics were collected from the underlying distributed storage layer (MinIO cluster). These metrics include request rates, resource utilization, storage usage, and node-level behavior during execution.

### 4.4. Backend Integration Using FastAPI

All the trained models are integrated into a single FastAPI application. Each of the tasks is provided with a unique API endpoint, enabling the processing of both structured data and MRI images using the same application backend.

Depending on the task, the API returns different types of results, such as:

- Classification outputs with probabilities and performance metrics (accuracy, precision, recall, F1-score)
- Forecasted KPI values along with regression error measures
- Predicted segmentation masks together with mIoU and Dice scores

**Figure 5:**
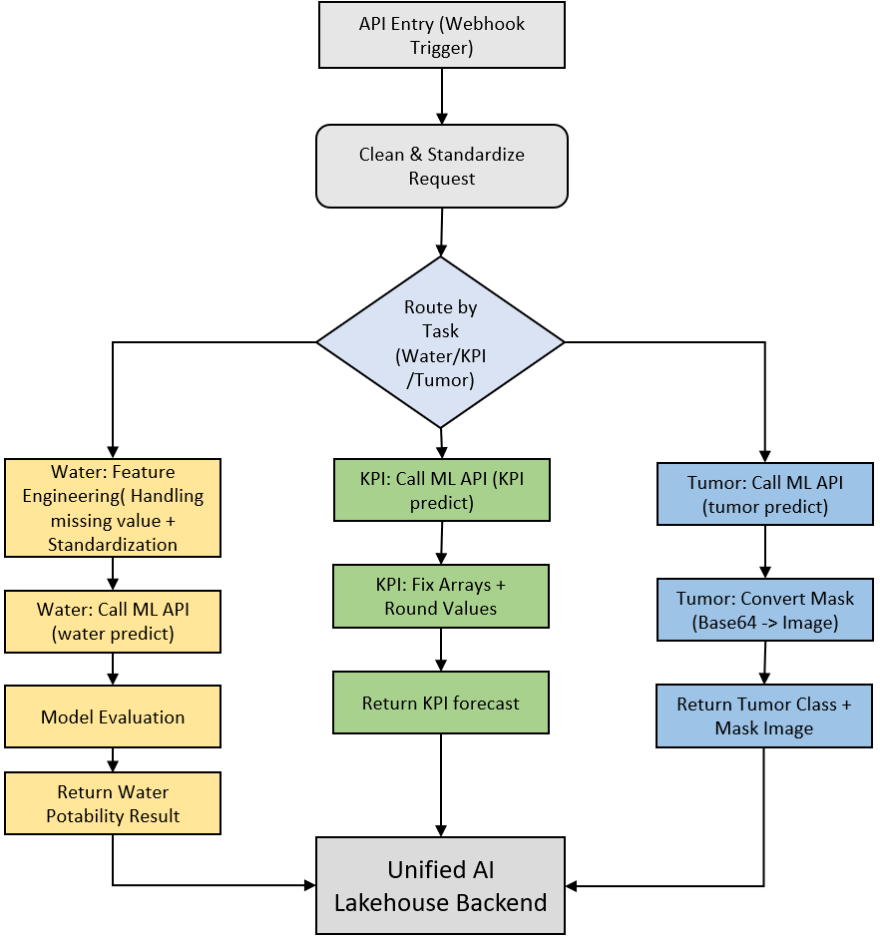
API-level workflow showing request routing and task-specific processing within the FastAPI backend.

The n8n workflow is used to route user requests to the correct endpoint and deliver the corresponding prediction results in a structured response, as shown in Figure 5.

### 4.5. Training Environment and Deployment

Model training was performed on a server at Lamar University due to higher computational power, especially for deep learning models. After training, the models were saved and integrated into the backend.

**Figure 6:**
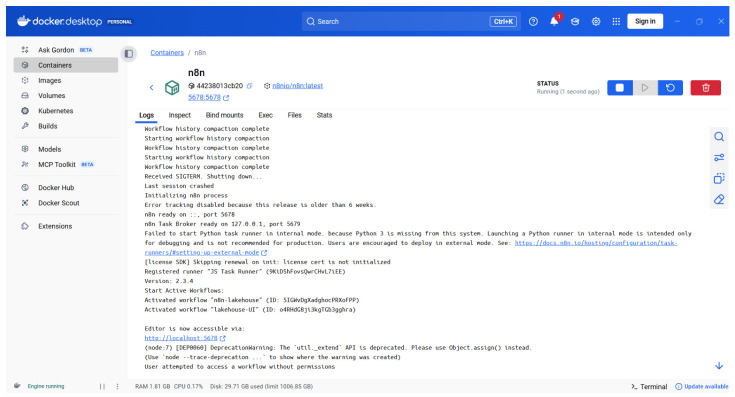
Container-based deployment structure enabling consistent execution across environments.

In this study, Docker is particularly used in the management of dependencies, which allows for reproducibility in executing the system, despite the machine used, thereby reducing variations, as shown in Figure 6.

### 4.6. Contribution of the Proposed Framework

Although Random Forest [2], ARIMA [3], and DeepLab-based segmentation [5] are well-known methods on their own, the key contribution of this work is bringing them together within a single backend system.

Instead of running different platforms for each individual task, this framework provides a single platform for medical image analysis, time series forecasting, and tabular prediction, all under a single umbrella, making the system simpler to handle and maintain.

## 5. Experimental Results and Comparative Analysis

### 5.1. Brain Tumor Classification Results

The classification model achieved consistently high performance across the 80/10/10, 70/10/20, and 60/10/30 evaluation settings. Test accuracy was 99.0%, 98.86%, and 98.91%, respectively, indicating strong generalization to unseen data under standard train–validation–test settings.

An additional robustness experiment was conducted using a smaller training subset (10/30/60 split). Under this setting, the test accuracy decreased to 95.44%, with similar reductions in precision, recall, and F1-score, suggesting that the model benefits from sufficient training data.

To further verify the correctness of the implementation, an extreme split (1/1/98) was evaluated, where only 1% of the data was used for training. In this case, the test accuracy dropped significantly to 74.89%, confirming that model performance depends heavily on the amount of available training data.

In addition to the image-level evaluation, patient-level classification experiments were conducted using a glioma MRI dataset from The Cancer Imaging Archive (TCIA) [15]. To keep the evaluation consistent, multiple train–validation–test splits were tested, including 80/10/10, 70/10/20, 60/10/30, and 10/30/60.

The confusion matrices indicate that most predictions are correct under standard settings, with only a limited number of misclassifications occurring mainly between visually similar tumor types such as meningioma and pituitary tumors. The detailed results across all splits are presented in Table 2.

Across these settings, the model achieved test accuracies between 70.07% and 76.92%, with F1-scores between 70.27% and 76.40%. Compared to the image-level results, the performance is lower, which is expected because all slices from a patient are kept within the same split. This makes the evaluation more realistic and removes any overlap between training and testing samples, as shown in Table 3.

The high classification accuracy observed under standard splits can be attributed to the clear visual differences between tumor classes in the MRI dataset, which enables ResNet50 to learn discriminative features effectively.

At the same time, the reduced performance observed under the 10/30/60 setting, and more significantly under the extreme 1/1/98 split, where the accuracy decreased to 74.89%, demonstrates that the model relies on sufficient training data to learn meaningful representations.

The patient-level results further support this observation, as the performance decreases when stricter splitting is applied. This confirms that the model is not relying on data leakage and can generalize to unseen patients under more realistic conditions.

### 5.2. Brain Tumor Segmentation Results

**Figure 7:**
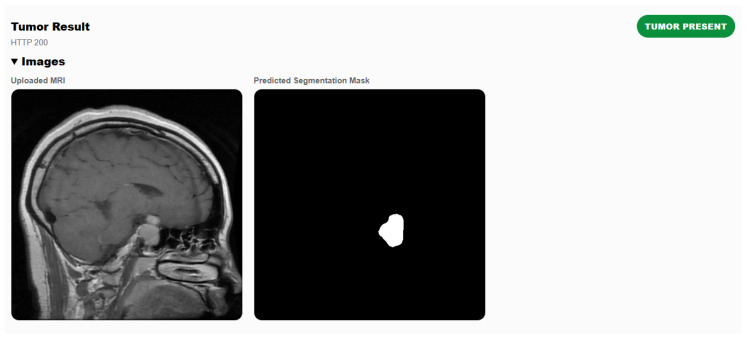
Example segmentation output showing input MRI and predicted tumor mask.

The segmentation model achieved 83.09% mIoU and 88.79% Dice score, indicating good performance in identifying tumor regions, as shown in Figure 7.

### 5.3. University KPI Forecasting Results

To address the concern regarding evaluation on interpolated data, two case studies were conducted. The first case study uses interpolated semester-level data to increase temporal resolution, while the second case study uses the original annual dataset without any modification. An 80/20 chronological split was applied consistently in both cases to ensure fair comparison.

KPI Case Study 1: Outcome Evaluation Using Interpolated Semester-Level Data

**Figure 8:**
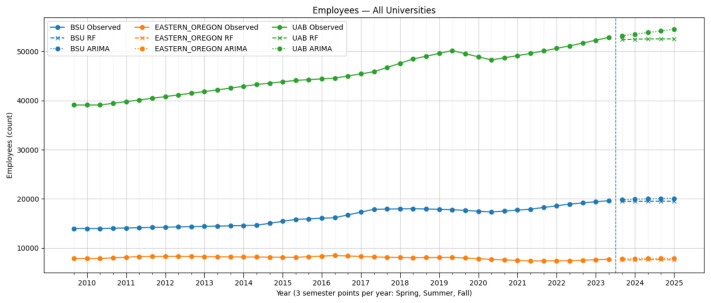
Employee forecasting comparison using interpolated semester-level data.

For employees, ARIMA showed strong and stable performance across all universities. The model achieved $R^{2}$ values of 0.9624 (BSU), 0.8153 (Eastern Oregon), and 0.9722 (UAB), with low MAE and RMSE values. In contrast, Random Forest produced negative $R^{2}$ values in all three cases, as shown in Figure 8.

**Figure 9:**
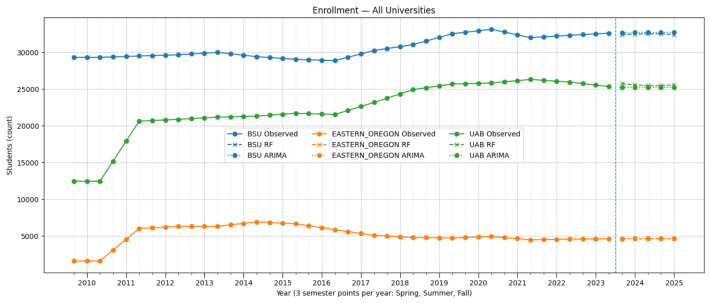
Enrollment forecasting comparison using interpolated data. The vertical dotted line indicates the boundary between the training and validation periods.

For enrollment, ARIMA again performed better than Random Forest; while BSU and Eastern Oregon remained difficult cases, ARIMA still achieved lower error values. UAB showed strong performance with $R^{2}=0.8326$, as shown in Figure 9.

**Figure 10:**
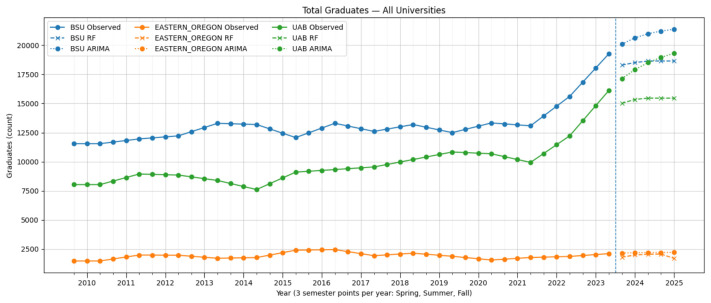
Graduates forecasting comparison using interpolated data. The vertical dotted line indicates the boundary between the training and validation periods.

For total graduates, ARIMA consistently outperformed Random Forest across all universities, achieving $R^{2}$ values above 0.85, as shown in Figure 10.

**Figure 11:**
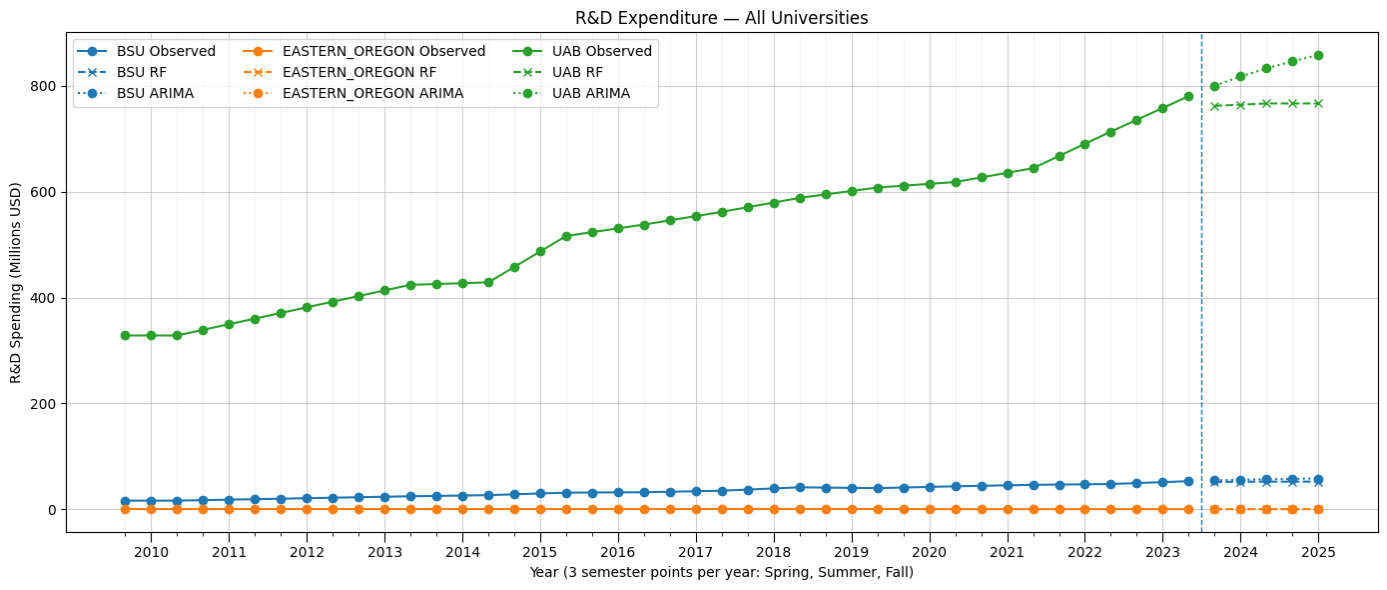
R&D forecasting comparison using interpolated data. The vertical dotted line indicates the boundary between the training and validation periods.

For R&D expenditure, ARIMA achieved strong performance for BSU and UAB, while Eastern Oregon remained unstable due to low variation, as shown in Figure 11.

KPI Case Study 2: Outcome Evaluation Using Original Annual-Level Data

When evaluated on the original annual dataset, the models showed noticeably weaker performance due to the limited number of observations.

**Figure 12:**
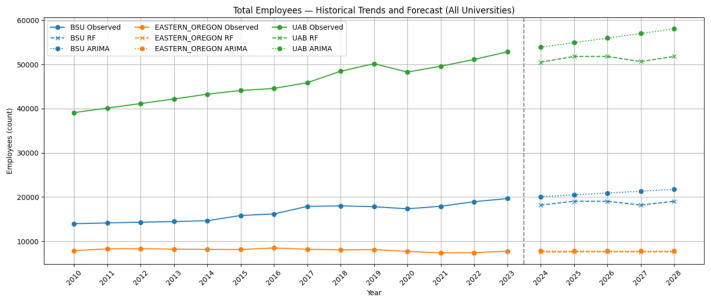
Employee forecasting comparison using original annual data.

For employees, ARIMA performance decreased, with $R^{2}$ values of 0.5690 (BSU), −1.3363 (Eastern Oregon), and 0.4653 (UAB). Random Forest remained unstable with negative $R^{2}$ values, as shown in Figure 12.

**Figure 13:**
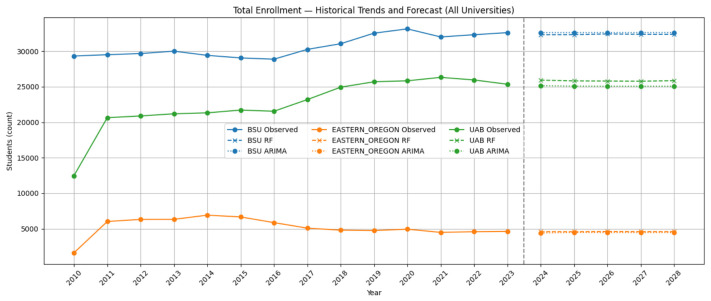
Enrollment forecasting comparison using original data. The vertical dotted line indicates the boundary between the training and validation periods.

For enrollment, all models struggled significantly. ARIMA produced negative $R^{2}$ values across all universities, indicating difficulty in learning meaningful patterns from limited data, as shown in Figure 13.

**Figure 14:**
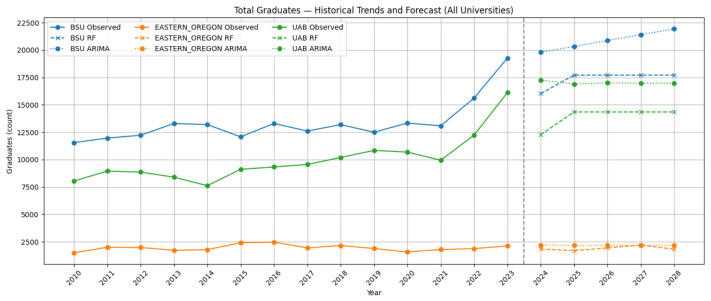
Graduates forecasting comparison using original data. The vertical dotted line indicates the boundary between the training and validation periods.

For total graduates, ARIMA showed reduced performance compared to the interpolated case, with mostly low or negative $R^{2}$ values, as shown in Figure 14.

**Figure 15:**
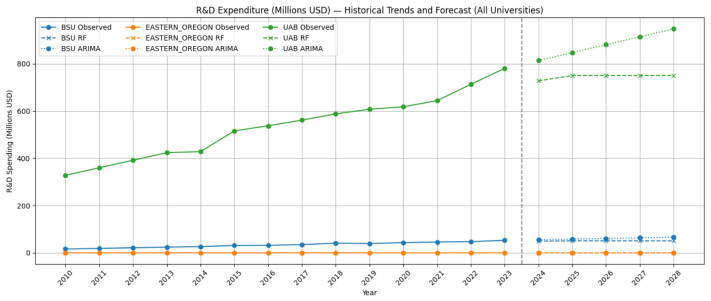
R&D forecasting comparison using original data. The vertical dotted line indicates the boundary between the training and validation periods.

For R&D expenditure, ARIMA remained relatively stable for BSU and UAB but performed poorly for Eastern Oregon, as shown in Figure 15.

Comparison of Both Case Studies

To provide a clearer comparison, Table 4 presents validation results for Boise State University (BSU) across both case studies.

Table 4 provides a detailed comparison using BSU as a representative example. The results show that ARIMA performs reasonably well on the interpolated dataset, with stable values across most KPIs. However, when evaluated on the original annual dataset, the performance drops noticeably, particularly for enrollment and graduates.

Random Forest shows less consistent performance in both cases and does not capture temporal patterns as effectively as ARIMA. The contrast between the two case studies indicates that interpolation makes the data smoother and easier to model, while the original dataset reflects the actual difficulty of forecasting with limited observations.

This comparison suggests that interpolation improves the apparent learning behavior of the models but does not introduce new information. Therefore, while interpolated data can be useful to demonstrate general trends, evaluation on the original dataset provides a more realistic understanding of model performance.

In practical settings, the reliability of forecasting models depends heavily on the availability of sufficient data. For short institutional time series, models like ARIMA can still provide reasonable estimates, but their performance remains limited when the dataset is small.

### 5.4. Case Study 3: Student Dropout Prediction Results

To evaluate the framework on a larger and more diverse dataset, an additional case study was conducted using a student dropout dataset [21].

A Random Forest classifier was trained using an 80/20 train-test split. Class weighting was applied to handle class imbalance and improve detection of dropout cases.

The model achieved the following performance on the test set, as shown in Table 5.

The results show that the model is able to identify dropout cases with improved recall, which is important in practical scenarios. Although the overall accuracy is slightly lower compared to balanced datasets, the use of class weighting improves the detection of minority class samples.

### 5.5. Water Potability Prediction Results

**Figure 16:**
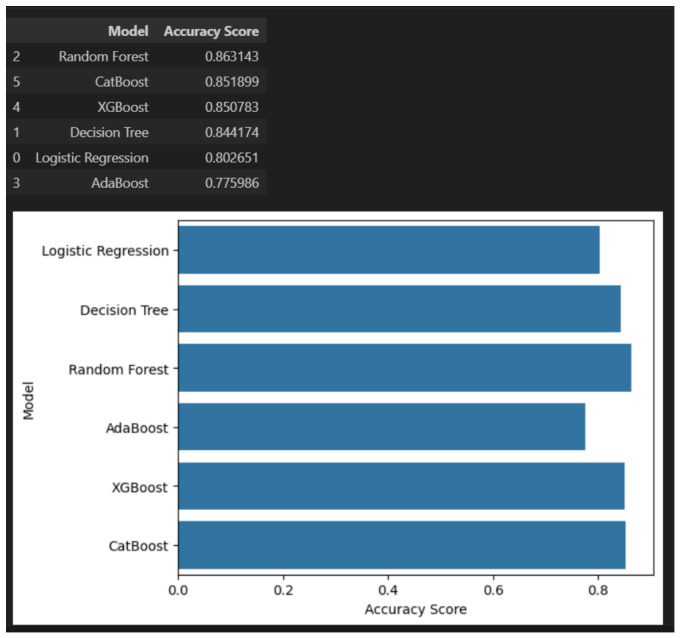
Comparison of classification accuracy across evaluated models for water potability prediction.

The water module was evaluated using an 80/20 train-test split. Multiple models were compared, and Random Forest achieved the best performance, as shown in Figure 16.

### 5.6. System-Level Benchmarking Results

System-level benchmarking was conducted to evaluate the runtime behavior of the framework under concurrent requests. The benchmark was performed for 60 s using 4 concurrent clients and 1 MiB objects.

Throughput and Request Rate: The system processed a total of 199 requests within the benchmark window, with an average rate of 49.8 requests per minute (approximately 0.83 requests per second).

Operation-Wise Performance: GET operations accounted for the largest portion of requests, followed by PUT, HEAD, and DELETE operations. The distribution of requests and corresponding rates are summarized in Table 6.

Latency: The time-to-first-byte (TTFB) for all operations remained below 50 ms, indicating consistent response time under concurrent load.

System Resource Utilization: Resource usage remained low during execution. CPU utilization ranged from 0.7% to 3.3% across nodes, and average memory usage remained below 500 MB per node, as shown in Table 7.

**Storage and Capacity:** The cluster maintained stable storage usage, with 60.5 GB used out of 599.7 GB total capacity (10.1% utilization), as shown in Table 8. This indicates that the system operates well within available resource limits.

The results show that the backend maintains stable request handling with low resource usage under moderate concurrent load.

## 6. Discussion

The brain tumor classification model based on ResNet50 achieved high performance under standard splits, with test accuracies between 98.86% and 99.0%. When the amount of training data was reduced (10/30/60), the accuracy dropped to 95.44%, and further decreased to 74.89% under the extreme 1/1/98 setting. This shows that model performance depends on the availability of sufficient training data.

Misclassifications were limited and mainly occurred between visually similar tumor types, such as meningioma and pituitary tumors. The overall performance can be attributed to the distinct visual patterns present in the MRI dataset.

For segmentation, metrics such as mIoU and Dice score provide a more meaningful evaluation than pixel accuracy due to the dominance of background regions in MRI images [10,12].

In addition to image-level evaluation, patient-level experiments were conducted using a glioma dataset from TCIA [15]. Across multiple split settings, the model achieved accuracies between 70.07% and 76.92%. The lower performance compared to image-level results reflects a more realistic scenario where all slices from a patient are kept within a single split, preventing data leakage.

The KPI forecasting results show that model performance is strongly affected by the size and structure of the data. The original dataset contains only 14 observations per university, which limits the effectiveness of models such as LSTM. Interpolation increases the number of data points but does not introduce new independent information.

Under these conditions, ARIMA performed more consistently than LSTM for short time-series data. The results also show that interpolation can lead to artificially improved performance due to smoothing, while evaluation on the original dataset provides a more realistic assessment.

The additional student dropout case study demonstrates that the proposed framework can also be applied to larger datasets. The results show that the model performs reasonably well under class imbalance, and the use of class weighting improves recall for dropout cases, which is important in practical applications.

Case Study 1, Case Study 2, and Case Study 3 together highlight the flexibility of the proposed framework. Case Study 1 uses interpolated KPI data to increase the number of observations, while Case Study 2 uses the original annual KPI data, which is limited and sparse. In contrast, Case Study 3 represents a larger and more structured tabular dataset.

The consistent performance across these cases shows that the proposed framework can handle different data sizes and structures, rather than being limited to a single type of dataset.

For water potability prediction, the Random Forest model showed balanced performance across metrics, particularly recall, which is important for identifying unsafe samples. Evaluating multiple metrics provides a more complete understanding of model behavior [30,31].

The main contribution of this work is not improving individual model accuracy, but demonstrating the integration of heterogeneous AI modules within a single backend system. The models are deployed using FastAPI and Docker, with workflow orchestration through n8n, enabling a unified interface for different tasks.

System-level benchmarking shows that the backend maintains stable request handling under concurrent load, with an average rate of approximately 49.8 requests per minute and time-to-first-byte below 50 ms across operations. Resource utilization remained low, with CPU usage below 3.3% and memory usage below 500 MB per node.

The current evaluation is performed under a fixed configuration. Further experiments with higher workloads and different deployment settings can provide additional insights into system scalability.

From a system design perspective, this work focuses on integration and deployment rather than comparing alternative architectures. A detailed analysis of scalability, fault tolerance, and resource efficiency is left for future work.

## 7. Limitations

The KPI forecasting module is based on annual institutional data. As a result, the time series are relatively short, which limits the effectiveness of models such as LSTM that typically require larger and more frequent observations.

The water potability model is trained using a large public dataset; while this supports reproducibility, the model may require region-specific data for deployment in real-world environmental monitoring scenarios.

Although a patient-level evaluation was conducted using an external glioma dataset from TCIA, the primary brain tumor classification dataset used in this study is image-based; while the additional experiment reduces the risk of data leakage and provides a more realistic assessment, future work will focus on fully patient-level datasets and multi-institutional validation to further strengthen generalization.

While the models were trained and tested using the Lamar University server, the system has not yet been deployed as a production-level service within an institutional environment. Therefore, further evaluation under real-world deployment conditions is required.

The KPI forecasting component is based on previous work and is included in this study as a case-study module to demonstrate system-level integration rather than to introduce a novel forecasting methodology.

## 8. Conclusions

This work presented an AI Lakehouse framework that integrates different AI workloads, including brain tumor MRI analysis, university KPI forecasting, and water potability prediction, within a single backend system. The implementation shows that models from different domains can be executed together in a shared environment.

For the brain tumor module, the classification and segmentation models performed well under the evaluated setup, showing that transfer learning can be effectively applied to medical image analysis. In addition to image-level evaluation, patient-level experiments were conducted using an external glioma dataset, which provided a more realistic assessment by ensuring that all slices from a patient were kept within a single data split. The observed performance under this setting reflects a more practical generalization scenario without data leakage.

For the KPI forecasting module, it was observed that model performance depends heavily on the amount and structure of the data. The interpolated dataset made the models easier to train, but evaluation on the original annual data showed that the performance drops due to the limited number of observations. This indicates that the KPI module should be seen more as a case-study component rather than a standalone forecasting contribution. Additionally, an extra case study using a student dropout dataset was conducted to evaluate the framework on a larger dataset and a different problem setting. The results demonstrate that the framework can be applied to classification tasks with imbalanced data, further supporting its applicability beyond the initial modules.

The water potability module showed consistent performance, especially in identifying unsafe samples, which supports its use for tabular prediction tasks.

From the system perspective, the benchmarking results show that the backend can maintain stable throughput and controlled latency under concurrent load. The benchmarking results also show balanced request distribution and low resource utilization across nodes during execution.

The main contribution of this work is demonstrating how different types of AI models can be integrated and deployed within a single backend system. The focus is on system design and practical deployment rather than improving individual models.

While the current evaluation was done under moderate workload conditions, testing under higher load and different deployment setups would help better understand system scalability.

## Figures and Tables

**Table 1:** Summary of related studies and identified research gap.

Study	Application Area	Approach	Research Gap
[4]	Image classification	ResNet	Backbone architecture; no integrated serving with other data types.
[5]	Image segmentation	DeepLabV3	Segmentation improvement; not integrated with tabular or forecasting systems.
[6]	Data platforms	Lakehouse architecture	Focuses on infrastructure; does not demonstrate integration of multiple AI model types.
[7]	Water quality	Neural networks	Applies ML to one domain only; no cross-domain deployment.
[8]	Water quality	ML comparison	Evaluates models but does not address system-level integration.
[9]	Time-series forecasting	LSTM	Introduces sequence model; not focused on multi-task deployment.
[10]	Medical imaging	BraTS benchmark	Provides evaluation dataset; no deployment framework.
**This Study**	Water + KPI + MRI	Integrated AI framework	Integrates tabular, time-series, and imaging models under one deployable backend system.

**Table 2:** Classification performance across different train–validation–test splits.

Split (Train/Validation/Test)	Accuracy	Precision	Recall	F1-Score
80/10/10	0.9900	0.9901	0.9900	0.9900
70/10/20	0.9886	0.9887	0.9886	0.9886
60/10/30	0.9891	0.9892	0.9891	0.9891
10/30/60	0.9544	0.9545	0.9544	0.9545
1/1/98	0.7489	0.7751	0.7489	0.7363

**Table 3:** Patient-level MRI classification performance across different train–validation–test splits.

Split	Accuracy	Precision	Recall	F1-Score
80/10/10	0.7692	0.7260	0.8063	0.7640
70/10/20	0.7380	0.6702	0.8884	0.7640
60/10/30	0.7414	0.7496	0.6613	0.7027
10/30/60	0.7007	0.6524	0.8260	0.7291

**Table 4:** Comparison of validation metrics for BSU using interpolated and original datasets.

KPI	Case Study	Model	*R* ^2^	MAE	RMSE	MAPE (%)
Employees	Interpolated	ARIMA	0.9624	111.67	140.51	0.61
Employees	Interpolated	RF	−0.7889	772.89	969.32	4.05
Employees	Original	ARIMA	0.5690	425.00	468.67	2.25
Employees	Original	RF	−2.7667	1183.00	1385.52	6.14
Enrollment	Interpolated	ARIMA	0.0038	162.56	225.92	0.50
Enrollment	Interpolated	RF	−0.1472	191.67	242.43	0.59
Enrollment	Original	ARIMA	−7.4847	531.33	717.21	1.65
Enrollment	Original	RF	−0.9556	319.33	344.33	0.99
Graduates	Interpolated	ARIMA	0.9424	432.67	515.47	2.76
Graduates	Interpolated	RF	−0.7373	2074.89	2830.39	12.13
Graduates	Original	ARIMA	−0.1480	2151.33	2717.16	12.14
Graduates	Original	RF	−1.6102	3218.33	4097.07	18.15
R&D	Interpolated	ARIMA	0.9656	0.32	0.50	0.66
R&D	Interpolated	RF	−0.7673	2.91	3.60	5.89
R&D	Original	ARIMA	0.6452	1.37	1.77	2.68
R&D	Original	RF	−5.5952	7.00	7.62	14.00

**Table 5:** Student dropout prediction performance.

Metric	Value
Accuracy	78.25%
Precision	53.17%
Recall	64.12%
F1-score	58.13%

**Table 6:** Operation-wise request performance.

Operation	Total Requests	Req/min	Request Share
GET (getobject)	89	22.25	44.7%
PUT (putobject)	51	12.75	25.6%
HEAD (headobject)	36	9.00	18.1%
DELETE (deleteobject)	23	5.75	11.6%
Total	199	49.75	100%

**Table 7:** Node-level resource utilization.

Node	CPU Usage (%)	Avg Memory (MB)	Max Memory (MB)
prd2	0.7	329.9	329.9
prd3	3.3	399.8	467.4
prd4	3.2	489.5	492.8
prd5	0.7	326.6	402.1

**Table 8:** Cluster storage utilization.

Metric	Value
Total Capacity	599.7 GB
Used Storage	60.5 GB
Usage Percentage	10.1%
Usable Free Space	269.7 GB
Objects Stored	1653

## Data Availability

The datasets used in this study are obtained from publicly available sources, including IEEE DataPort, TCIA, IPEDS, NSF HERD, and Kaggle, as cited in the manuscript. All preprocessing steps are described in the manuscript. Processed files used in this work are available from the corresponding author upon reasonable request. The datasets used in this study are publicly available and are used only for research and educational purposes. The authors (mainly first author) are responsible for the analysis, coding, and interpretation presented in this manuscript.

## Supplementary Materials

The following supporting information can be downloaded at: https://www.mdpi.com/article/10.3390/s26123804/s1, Table S1: Processed semester-level KPI dataset for Boise State University (BSU), Eastern Oregon University (EOU), and the University of Alabama at Birmingham (UAB), generated through linear interpolation from IPEDS and NSF HERD annual data (2010–2023).
